# Genomic Distribution and Inter-Sample Variation of Non-CpG Methylation across Human Cell Types

**DOI:** 10.1371/journal.pgen.1002389

**Published:** 2011-12-08

**Authors:** Michael J. Ziller, Fabian Müller, Jing Liao, Yingying Zhang, Hongcang Gu, Christoph Bock, Patrick Boyle, Charles B. Epstein, Bradley E. Bernstein, Thomas Lengauer, Andreas Gnirke, Alexander Meissner

**Affiliations:** 1 Broad Institute of Harvard and MIT, Cambridge, Massachusetts, United States of America; 2 Department of Stem Cell and Regenerative Biology, Harvard University, Cambridge, Massachusetts, United States of America; 3 Harvard Stem Cell Institute, Cambridge, Massachusetts, United States of America; 4 Max Planck Institute for Informatics, Saarbrücken, Germany; 5 Howard Hughes Medical Institute, Chevy Chase, Maryland, United States of America; 6 Department of Pathology, Massachusetts General Hospital and Harvard Medical School, Boston, Massachusetts, United States of America; 7 Center for Systems Biology and Center for Cancer Research, Massachusetts General Hospital, Boston, Massachusetts, United States of America; Friedrich Miescher Institute for Biomedical Research, Switzerland

## Abstract

DNA methylation plays an important role in development and disease. The primary sites of DNA methylation in vertebrates are cytosines in the CpG dinucleotide context, which account for roughly three quarters of the total DNA methylation content in human and mouse cells. While the genomic distribution, inter-individual stability, and functional role of CpG methylation are reasonably well understood, little is known about DNA methylation targeting CpA, CpT, and CpC (non-CpG) dinucleotides. Here we report a comprehensive analysis of non-CpG methylation in 76 genome-scale DNA methylation maps across pluripotent and differentiated human cell types. We confirm non-CpG methylation to be predominantly present in pluripotent cell types and observe a decrease upon differentiation and near complete absence in various somatic cell types. Although no function has been assigned to it in pluripotency, our data highlight that non-CpG methylation patterns reappear upon iPS cell reprogramming. Intriguingly, the patterns are highly variable and show little conservation between different pluripotent cell lines. We find a strong correlation of non-CpG methylation and DNMT3 expression levels while showing statistical independence of non-CpG methylation from pluripotency associated gene expression. In line with these findings, we show that knockdown of DNMTA and DNMT3B in hESCs results in a global reduction of non-CpG methylation. Finally, non-CpG methylation appears to be spatially correlated with CpG methylation. In summary these results contribute further to our understanding of cytosine methylation patterns in human cells using a large representative sample set.

## Author Summary

Epigenetic modifications including DNA methylation at the position 5 of the cytosine base provide regulatory information to the genome sequence. The primary target of cytosine methylation in mammals is the CpG dinucleotide. However, previous studies in the mouse and more recent work in humans have highlighted the presence of non-CpG methylation in pluripotent cells. Currently, little is known about the role of this type of DNA methylation. We sought to further characterize non-CpG methylation by employing a comprehensive data set of genome-scale methylation maps across various human cell types. Our analysis reveals that non-CpG methylation varies dramatically between pluripotent cells and is closely linked to CpG methylation. Moreover, we show that depletion of the *de novo* DNA methyltransferases results in a global reduction of non-CpG methylation levels. Taken together, these findings further advance our understanding of cytosine methylation and describe its distribution among a large number of human cell types.

## Introduction

DNA methylation as a regulatory epigenetic mechanism is a widespread phenomenon [1]. In vertebrates, the CpG dinucleotide is the predominant target for methylation. However, several murine studies have shown the presence of non-CpG methylation in ES cells [2] and early embryos [3], but its near complete absence in somatic tissues [4]. In contrast plants exhibit frequent non-CpG (CpNpG and CpHpH) methylation and have established mechanisms to propagate CpNpG and asymmetric CpHpH methylation marks. These differences between plants and mammals can partly be attributed to the presence of distinct methyltransferases. While *A. Thaliana* possess specific DNA MethylTransferase (DNMT) classes that exhibit a strong sequence preference for either CpG dinucleotides or CpHpG trinucleotides, mammalian cells lack the latter class of Chromomethylase DNA methyltransferases [5]. Instead, there are only three mammalian DNA methyltransferases exhibiting significant catalytic activity on DNA [6]. These enzymes show a strong preference for CpG dinucleotides. However, the murine *de novo* methyltransferases Dnmt3a and Dnmt3b also facilitate methylation of cytosines in non-CpG context at a rate 40–500 fold below the CpG levels [2], [6]. Ectopic expression of murine Dnmt3a in *D. melanogaster* suggested that this enzyme is capable of *de novo* methylation that includes also non-CpG targets [2]. In line with these studies, it has been shown that mES cells exhibit detectable levels of non-CpG methylation and express Dnmt3a and Dntm3b at higher levels than most somatic cell types [2], [7]. A role for Dnmt3a or Dnmt3b in establishing non-CpG methylation is further supported by studies of Dnmt3a and 3b double knockout mES cells that at early passages showed dramatic reduction in non-CpG, but not CpG, methylation levels globally and in newly integrated retroviruses [7], [8]. In contrast, Dnmt1 KO mES cells exhibit dramatic loss of CpG methylation while non-CpG methylation levels were not affected [2]. Therefore the involvement of DNMT1 in the establishment or maintenance of non-CpG methylation seems limited. In line with the murine findings, three studies involving whole genome bisulfite sequencing of human embryonic stem cells (hESCs) and fibroblasts reported significant levels of non-CpG methylation in stem cells [9]–[11] amounting to approximately 25% of all methylated cytosines. When calculated against all non-CpG dinucleotides, this corresponds to an average non-CpG methylation of 1.3% compared to 55–80% for CpG methylation [9], [10]. In addition, a dramatic reduction of non-CpG methylation frequency in two somatic cell types, fibroblasts and monocytes, was reported suggesting the confinement of this phenomenon to the pluripotent state. A recent study reported several megabase regions that failed to reestablish non-CpG methylation patterns in human induced pluripotent stem cells (iPSCs) [11]. However, given the limited murine studies and the still small number of available human pluripotent cell methylomes, it remains unclear what the extent, relevance and inter-sample variation of non-CpG methylation is.

To systematically address these questions, we analyzed cytosine methylation in a number of published data sets [12] in combination with 30 new, unpublished genome-scale DNA methylation maps (Table S1). This large pool of samples enabled us to characterize non-CpG methylation in a total of 76 data sets from pluripotent (ES and iPS), pluripotent cell derived and somatic cells. These results show non-CpG methylation – contrary to CpG methylation - to be a highly variable and rare phenomenon. While CpA methylation is by far the dominant form of non-CpG methylation in pluripotent cell types, non-CpG methylation in the somatic cells that we investigated is approximately equally distributed at background levels among CpA, CpT and CpC. We show that this decrease of non-CpG methylation occurs relatively early upon initiation of differentiation coinciding with the down-regulation of DNMT3A and DNMT3B gene expression. Our bioinformatics results suggest that DNMT3, rather than pluripotency gene expression levels are highly predictive of non-CpG methylation levels. In further support of this, we demonstrate that stable knockdown of the *de novo* DNMTs leads to a global reduction in non-CpG methylation with no effect apparent effect on pluripotency gene expression. On the sequence level we observe CpA methylation to be highly correlated with the presence of methylated CpGs in close vicinity. Our findings provide a more comprehensive understanding of the so far sparsely characterized non-CpG methylation.

## Results

### Efficient detection of non-CpG methylation by Reduced Representation Bisulfite Sequencing (RRBS)

**Figure 1:**
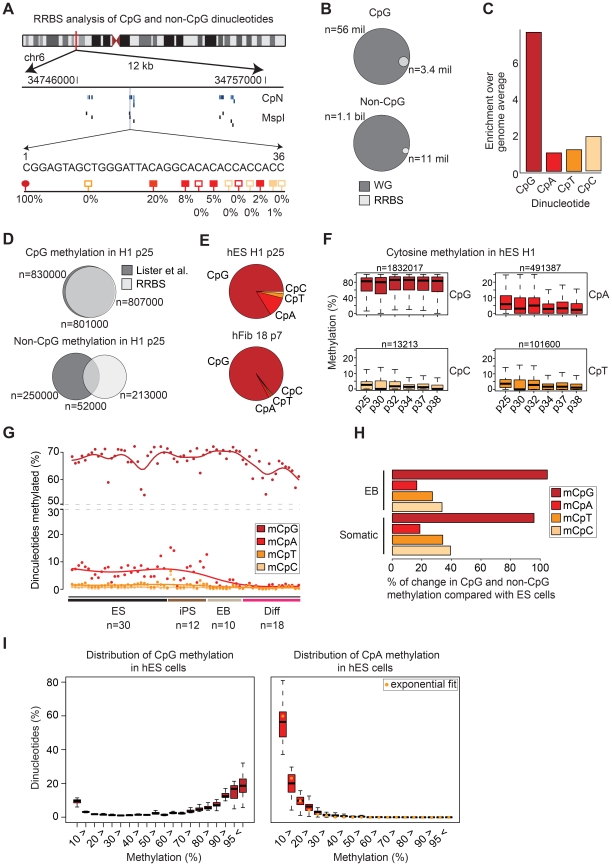
Global distribution of CpG and non-CpG methylation in human cell types. (A) Schematic of RRBS data visualization and a selected 36 bp read. Blue lines indicate covered cytosines (CpN), black lines MspI restriction sites (middle). One selected RRBS read in this region is shown (bottom). Red circles indicate CpGs, light red boxes CpTs, dark red boxes CpAs and yellow boxes CpCs. Filled circles and boxes indicate dinucleotides with detectable levels of methylation. The percent below indicate the methylation levels by averaging the methylation state of a given cytosine over all reads that cover its position. (B) Venn diagrams show the theoretical RRBS coverage compared to the whole genome for CpGs (top) and non-CpGs (bottom) based on a 40–260 bp size selection. (C) Enrichment of cytosine dinucleotide frequency for RRBS relative to the whole genome. (D) Venn diagrams show the overlap of methylated CpGs (top) as well as methylated non-CpGs (bottom) exhibiting above threshold (≥10% and ≥5%) methylation in the whole methylome (WM) data by Lister *et al.* 2009 and our RRBS data for the same cell line and passage. Only those dinucleotides were considered that were covered in both data sets simultaneously by at least 5 reads. Numbers below the venn diagrams indicate overlap of both dinucleotide sets. (E) Pie chart of sequence context distribution of methylated cytosines in the human ESC line H1 (passage 25) and human fibroblasts 18 (passage 7). (F) Boxplots of the methylation levels as assessed by RRBS across six biological replicates of hESC line H1. Boxplots are based on all cytosine dinucleotides that show any evidence for methylation in H1 (median methylation ≥0.1% over all six replicates). Boxes are 25^th^ and 75^th^ quartiles, whiskers indicate most extreme data point less than 1.5 interquartile range from box and black bar represents the median. n indicates the number of dinucleotides covered in all and methylated in at least one of the six samples. (G) Distribution of methylated (≥10%) cytosine dinucleotides in human ES cells (ES, n = 30), iPS cells (n = 12), embryoid bodies (EB, n = 10) and 10 somatic cell types (n = 18). Percentages are methylated cytosine dinucleotides divided by corresponding total number of each cytosine dinucleotide with ≥5x coverage. (H) Barplot showing the average reduction in the number of methylated cytosine dinucleotides in EBs (n = 10) and somatic cells (n = 18) relative to pluripotent cells (n = 42). (I) Distribution of distinct CpG (left) and CpA (right) methylation levels for all CpA and CpG dinucleotides averaged over all hES samples (n = 30). The medians of the CpA methylation level distribution are fitted by the exponential distribution (yellow circle). Boxplots are defined as in (F).

The most comprehensive maps of non-CpG methylation in human cells to date have been generated using whole genome bisulfite sequencing [9], [10], [13]. Like methylC-seq [9], [10], [13] RRBS is based on bisulfite conversion and capable of detecting CpG and non-CpG methylation (Figure 1A). However, due to its design to enrich for CpG rich regions, we first wanted to assess our method's ability to measure non-CpG methylation in a representative fashion. Using a standard 36 bp single-end sequencing protocol, RRBS enables the investigation of around 3.4 million (6.1% of all) CpG dinucleotides and about 11.5 million (1.03% of all) non-CpG dinucleotides when employing a size selection of 40–260 bp (Figure 1B; Table S1). The RRBS protocol applies the methylation-insensitive restriction enzyme *Msp*I and thus is by design biased towards CpG richer regions in the genome (Figure 1C) while still providing representative coverage of many key genomic features (Figure S1A). For the other three dinucleotide combinations, there is either no detectable bias (CpA, CpT) or a 2-fold enrichment (CpC) (Figure 1C). Comparative analysis of previously published whole genome bisulfite sequencing data [9] and our RRBS data for the hESC line H1 demonstrates that the overlap of CpGs that are contained in both data sets and designated methylated is substantial (Figure 1D, top). In addition, a significant fraction of methylated non-CpGs reported earlier [14] are also captured using RRBS (Figure 1D, bottom). Methylated non-CpGs in the whole methylome and RRBS data from the same DNA (H1 p25 [14]) exhibit an overlap of approximately 32% (p<2.2e-16 Fisher exact test, Figure 1D). Notably, this observation is in line with the overlap of non-CpG methylation between the two whole methylome replicates previously reported [9]. In order to further validate the capacity of RRBS to capture non-CpG methylation, we compared the spatial distribution of CpG and CpA methylation levels across key genomic features to whole genome bisulfite sequencing (WGBS) data for hESCs generated in our laboratory. This comparison provides additional evidence that RRBS is capable of recapitulating genome-wide methylation trends in CpG and non-CpG methylation (Figure S1D). Finally, we confirmed our observations of elevated CpA methylation levels in our RRBS data by locus-specific bisulfite sequencing (Figure S2). Taken together, these results demonstrate that RRBS is suitable to accurately capture a small, but representative fraction of non-CpGs throughout the genome.

### Non-CpG methylation is predominately found in pluripotent cells

We have selected 70 RRBS data sets of pluripotent and differentiated cells for our initial analysis. This data set comprises 32 distinct pluripotent lines (20 ESC and 12 iPSC lines; 42 samples in total that include different passage numbers of the same lines) and 20 distinct differentiated samples. These include 10 ESC or iPSC derived embryoid bodies (EB) and 10 somatic cell types or tissues (Table 1 and Table S1), adding up to n = 52 distinct cell or tissue types and 70 samples in total. A detailed summary of all the samples, their bisulfite conversion rates and which data sets have been previously published is provided in Table S1 (all data are publically available through the NIH Roadmap Epigenomics Project: http://www.roadmapepigenomics.org/).

**Table 1:** Summary statistics for samples included in this study.

Sample name	uniqueSeqMotifCount (million)				%mCN/CN			
	CpG	CpA	CpC	CpT	CpG	CpA	CpC	CpT
HUES (n = 30)	3.16	3.70	5.19	4.43	67.85%	6.68%	0.63%	1.48%
iPS (n = 12)	3.07	3.63	5.17	4.42	68.31%	7.81%	1.05%	1.99%
EB (n = 10)	3.24	3.70	5.03	4.34	70.40%	1.74%	0.35%	0.57%
NPC	3.34	3.66	5.55	4.78	57.04%	0.35%	0.18%	0.18%
Pancreatic islet	3.06	3.32	4.94	4.13	61.15%	1.52%	0.17%	0.40%
Fibroblasts (n = 6)	3.10	3.59	5.09	4.33	66.52%	1.09%	0.68%	0.74%
Rectal mucosa	2.89	2.93	4.20	3.60	60.59%	0.18%	0.05%	0.05%
Rectal smooth muscle	2.76	2.67	3.96	3.35	54.64%	0.34%	0.05%	0.10%
Skeletal muscle (n = 2)	2.90	2.93	4.20	3.62	62.90%	1.70%	0.15%	0.43%
Stomach muscle	2.91	2.96	4.18	3.60	62.13%	0.30%	0.04%	0.08%
Blood CD19	3.25	3.78	5.59	4.75	66.79%	1.36%	0.74%	1.00%
Blood CD34 (n = 2)	3.25	3.77	5.66	4.79	65.30%	1.65%	0.59%	0.89%
Whole Blood (n = 2)	1.51	1.67	2.14	1.97	63.85%	1.40%	0.74%	1.04%

Sample categories with corresponding sample number n and median number of distinct cytosine dinucleotides covered. In addition, the percentage of methylated cytosines (≥10%) covered by ≥5x is shown for each cytosine dinucleotide category (%mCN/CN).

Starting with the H1 (passage 25) ESCs [14], we found that among the three possible non-CpG dinucleotides (CpA, CpT and CpC) that CpA methylation is the most frequent (∼12%), followed by CpT (∼2.6%) and CpC (1.2%) methylation (Figure 1E). These ratios are consistent with previous non-bisulfite based reports in the mouse [2], [8]. To investigate this further we picked six different passages of the H1 ESCs and calculated the methylation levels of the four dinucleotides (Figure 1F). As before, most CpGs are methylated, while CpA shows substantially lower levels followed by CpT and CpC. The total number of CpT sites that show above threshold methylation levels (see Materials and Methods) in any one of the H1 samples is only 101,600. CpC methylation occurred at an even lower frequency than CpT methylation, constituting only 13,213 sites across the six H1 samples (Figure 1F). Furthermore, CpC methylation levels were also not correlated across multiple samples (Figure S3A), suggesting that the majority of CpC methylation more likely to be an artifact of bisulfite conversion. We next compared the levels among all 70 data sets and found that consistent with previous studies, we observe a dramatic decrease of non-CpG methylation in somatic cell types (Figures 1G, 1H). In contrast to pluripotent cells, most of the somatic cell types included in this study show almost complete absence of non-CpG methylation. Some, but not all, day 16 EBs retain slightly higher, though clearly reduced non-CpG methylation levels (Figure 1G). In pluripotent cells (n = 42), cytosines in the non-CpG contexts on average constituted 12.7% to all methylated cytosines. Though slightly lower this number is overall consistent with previous reports using whole methylome data [9], [10]. The differences might be attributed to the fact that RRBS enriches for CpG dense regions including CG islands (which are mostly unmethylated) or alternatively could be caused by differing conversion rates between the previous studies and our data sets here.. On average, across all our pluripotent cell lines, approximately 9.3% of all methylated cytosines occur in the CpA context, corresponding to approximately 6.8% of all CpAs covered (Figure 1G). In contrast, more than 85% of methylated cytosines occur in the CpG contexts indicating that about 68.1% of covered CpGs are methylated (Figure 1G).

To better understand the dynamics as well as the sensitivity of RRBS to detect these changes, we investigated the reduction of non-CpG methylation in ESC/iPSC derived EBs (n = 10) and somatic cells (n = 18) compared to pluripotent cells (n = 42). Clearly, CpA and CpT methylation experience the most dramatic change with reduction by 85% and 80% respectively (Figure 1H). CpC methylation is also reduced, but only decreases by 65% in both EBs and somatic cells. The difference in reduction of methylation levels in combination with comparable methylation abundance of all non-CpG sequence contexts in differentiating and somatic cells might allow for a conservative assessment of the noise level (incomplete conversion) in methylation measurements. Notably, the intermediate non-CpG methylation levels in a subset of differentiating cells (EBs) show that RRBS is capable of detecting even subtle differences in non-CpG methylation (Figure 1G, 1H).

In addition to the striking difference in overall abundance, the distribution of methylation levels are quite distinct between non-CpGs and CpGs (Figure 1I, shown only for CpG and CpA) [9]. As reported previously, CpG methylation levels follow a bimodal distribution in nearly all cell types [9], [15], [16] with the two means representing low (<10%) and high (>85%) methylation levels (Figure 1I, left panel). In contrast, CpA methylation levels follow an exponential distribution with a very narrow tail towards high methylation levels (Figure 1I, right panel). In combination with the low overall number of CpAs showing methylation it suggests that CpA methylation is a rare event. As noted above, CpT and CpC methylation occur at even lower frequencies than CpA methylation and are subject to a high level of noise. Since the analysis of CpT and CpC did not provide any additional insights we decided to focus the data presentation on CpG and CpA methylation comparisons.

### CpA methylation exhibits little conservation across several passages

Before comparing CpA methylation levels across different samples we wanted to test whether any particular regions of the genome possess enrichment for non-CpG methylation. Global investigation of the distribution across several genomic features revealed no particular hot spots (Figure S3B). We next analyzed CpA methylation in 20 ES cells lines (30 data sets) on more than 1.7 million consensus CpAs (defined as having ≥2x coverage in ≥80% of the samples). Consistent with previous reports [9], [10], CpA methylation levels vary dramatically between samples (Figure 1G, Figure S3C) while individual CpG methylation levels are more robust (Figure 1G, Figure S3D).

**Figure 2:**
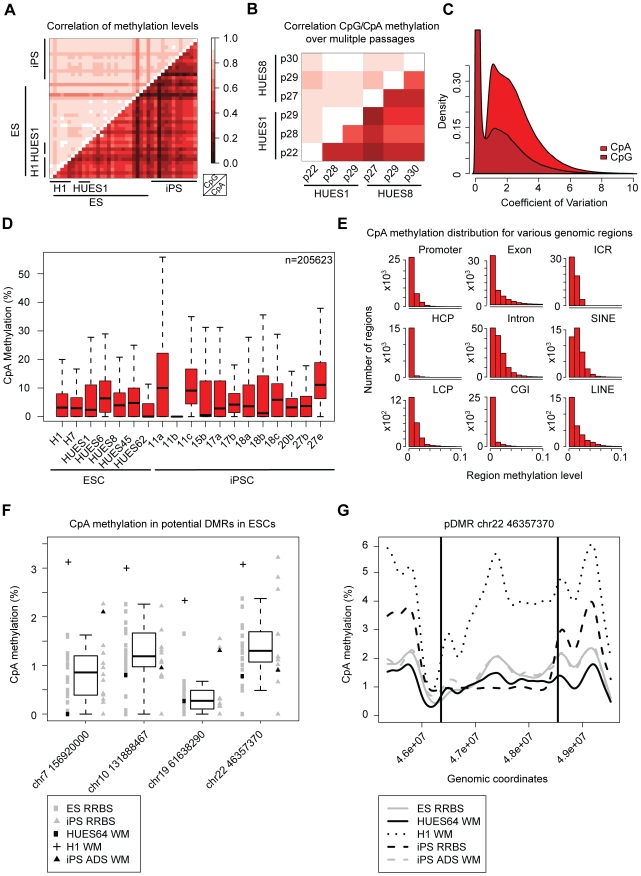
CpA methylation shows little conservation over several passages. (A) Heatmap of pearson correlation coefficients for CpG (upper triangle) and CpA (lower triangle) methylation patterns in all pairs of pluripotent cell lines. Selected lines are highlighted. (B) Heatmap showing the pearson correlation coefficients for CpG (upper triangle) and CpA (lower triangle) methylation levels in pairs of pluripotent cell lines assessed at consecutive passages. (C) Distribution of the coefficient of variation over all individual CpG and CpA methylation levels across all ESC samples (n = 30). (D) Boxplot of CpA methylation levels in 7 ESC and 12 iPSC lines. Boxplots are based on 205623 CpAs that show more than 0.1% of median methylation in the selected ESC lines (n = 7). Boxes are 25^th^ and 75^th^ quartiles, whiskers indicate most extreme data point less than 1.5 interquartile range from box and black bar represents the median. (E) Distribution of CpA methylation levels in different genomic region classes averaged over a representative set of pluripotent cell lines at different passages (n = 12: H1, HUES1, HUES3, HUES6, HUES8, HUES45, H9, iPS 15b). HCPs are defined as promoters overlapping with a CG island, LCPs are promoters without a CG island. For a detailed definition of the regions see Materials and Methods. (F) Boxplot of CpA methylation levels across four genomic regions over all distinct ESC lines (n = 20) assessed by RRBS. These regions were reported to be consistently hypomethylated between five iPSC and two ESC lines [11]. In addition methylation levels from previously published whole genome bisulfite sequencing (WM) for H1 [9], iPSC ADS [11] as well as our HUES64 WM are shown. Boxes are 25^th^ and 75^th^ quartiles, whiskers indicate most extreme data point less than 1.5 interquartile range from box and black bar represents the median. (G) CpA methylation profile of one selected DMR (framed by black lines) on chromosome 22 based on a 1 kb tiling. The CpA methylation levels based on RRBS are shown for the median of all ESCs (n = 20) and all iPSCs (n = 12) as well as WM levels for H1p25, iPS ADS and HUES64.

The impact of this variation becomes even more evident when considering the conservation of CpA methylation between different samples: While the average Pearson correlation coefficient (PCC) between different ES cell lines is about r = 0.37 for CpA methylation (Figure 2A, lower triangle), it is greater than r = 0.90 for CpG methylation (Figure 2A, upper triangle). To assess the levels of CpA methylation over several passages we used six biological replicates of the H1 ESCs as well as biological replicates from HUES8 and HUES1. Notably, CpA methylation levels in the H1 samples yield an average PCC of only r = 0.35. Interestingly, the variation in CpA methylation between different ES cell lines is about as large as the variation between different passages of the same cell line. However, two specific cell lines that are just one passage apart (HUES8 p29 and p30 and HUES1 p28 and p29) had slightly higher correlation (r = 0.54), while further increasing the passage number reduced the correlation to average levels (r = 0.36, Figure 2B, lower triangle). In contrast, CpG methylation levels in samples from the same ESC line at different passages generally exhibit the lowest fluctuations (Figure 2A, 2B; upper triangles). These findings are further highlighted by the strong variation in methylation levels that individual CpAs exhibit over the 30 ES cell samples. While the average coefficient of variation is less than 0.5 for CpGs, it is around 3 for methylated CpAs (Figure 2C). These observations highlight the variability in levels of CpA methylation.

### Non-CpG methylation patterns are similar in ESC and iPSCs

It has been previously shown that non-CpG methylation patterns reappear upon reprogramming of somatic cells to iPSCs [9], [11]. To further expand on this we determined CpA methylation levels in 12 iPSC lines comprising passage numbers ranging from 14 up to 44 (Figure 2D, Table S1) [17]. Overall, iPSCs exhibit CpA methylation levels and patterns comparable to ESCs (Figure 2A, 2D), confirming previous reports [11]. Only two iPSC lines in our set, 11a and 27e, showed slightly elevated CpA methylation levels. We next asked, whether these differences in CpA methylation levels are associated with specific genomic features. To this end, we determined the average levels across features using the six samples of the H1 ESCs and the two iPSC lines that exhibited elevated CpA methylation levels (11a and 27e). While no specific region class exhibited particularly elevated levels of CpA methylation, introns and SINE repeats showed the highest CpA methylation levels (Figure 2E). In contrast, CpG island promoters and CpG islands were depleted of CpA methylation. The latter finding is consistent with the generally observed low CpG methylation levels in high CpG promoters (HCPs) and CpG islands [15] (Figure S3F) and higher methylation levels in CpG poorer regions. The comparison of the average CpA methylation levels in the H1 samples to the iPSC lines 11a and 27e revealed that the elevated CpA methylation levels in the two iPS cell lines did not affect any unique region class. Instead all region classes exhibited higher methylation levels. These observations are in line with previous reports, confirming no broad differences in terms of DNA methylation between ESC and iPSCs [11], [12]. Despite the overall similarity, it has been observed that iPSCs exhibit distinct non-CpG methylation patterns in specific genomic regions (hypomethylated DMRs) compared to the ESC lines H1 and H9 [11]. To assess whether this is a more general phenomenon, we took advantage of our 20 ESC and 12 iPSC lines and investigated four non-CpG DMRs reported in the previous study [11] that had representative RRBS coverage. While we find that some iPSC lines show reduced methylation levels in multiple of these regions compared to the ESC methylation distribution, the majority of the iPSCs clearly resemble the CpA methylation pattern of ESCs within these large blocks (Figure 2F). Moreover, several ESC lines also exhibit reduced CpA methylation levels compared to the RRBS based ESC average (Figure 2F, black bar indicates the median of all 20 ESC lines).

As an additional independent confirmation we selected a small subset of samples (n = 8) and investigated their CpG and non-CpG patterns using the Illumina Infinium HumanMethylation450 array [18]. The array captures the methylation status of approximately 482,000 CpG dinucleotides and 3200 non-CpG dinucleotides. We found the median methylation levels of all CpGs covered by the array to be slightly lower than measured by RRBS with an average of 70% (Figure S4A, Figure S3D and S3E) and found little variation between this subset of samples. In contrast, non-CpG methylation levels varied by more than 15% between the samples regardless of whether they are ESCs or iPSCs. Notably, median non-CpG methylation levels lay between 30% and 50% (Figure S4B), which is on average more than 30% higher compared to RRBS measurements (Figure 2D, Figure S3D). Interestingly, the trend in total non-CpG methylation levels is conserved between the array and RRBS data: Samples exhibiting high non-CpG methylation levels according to the array show also elevated CpA methylation levels according to the RRBS data and *vice versa* (Figure S4B, Figure 2D, Figure S3D). To compare the Infinium array and RRBS results more quantitatively, we restricted our subsequent analysis to genomic regions that can be queried by both methods. Given the relatively small number of sites on the array it is not surprising that the direct overlap between the array and RRBS is only moderate for CpG and non-CpG sites (Figure S4C, S4D). We had previously shown that methylation levels for individual CpGs on the Infinum platform were generally well correlated with their neighboring sites [19]. To increase the number of dinucleotides that can be compared between RRBS and the array we applied a similar approach here. The median number of regions around Infinium probes (100 bp on either side) sufficiently covered by RRBS was 185,658 for CpG and 60 for CpA probes. The restriction to this set of cytosines yields good agreement for CpG methylation levels measured by RRBS and Infinium (Figure S4E). In contrast, non-CpG methylation exhibits only little correlation between RRBS and the Infinium array (Figure S4F) while non-CpG methylation is well correlated among the Infinium samples. It is important to note that CpG methylation has been shown to be consistent in regions thereby allowing the above analysis, whereas non-CpG methylation appears at single loci in part explaining the lower correlation. In conclusion, RRBS inferred methylation levels of CpGs are in excellent agreement with the Infinium assay whereas due to small overlap and biased selection of probes on the array CpA methylation does not exhibit consistency between both methods.

To extend our RRBS based findings on the putative non-CpG DMRs, we also included the previously published data that were used to identify them, i.e. whole methylome (WM) data for H1 passage 25 [9] and ADS iPS cells [11] as well as our HUES64 WM data. To ensure comparability of all data sets, we reprocessed the raw data for H1 WM and ADS iPS WM using our own alignment and analysis pipeline. This extended comparison revealed high concordance between RRBS, the ADS iPS WM and the HUES64 WM in putative DMRs. Both the ADS iPS WM and the HUES64 WM fall within the reference corridor established by the 20 RRBS ESCs profiles (Figure 2F). In contrast, the H1 WM shows significantly increased methylation in the four putative DMRs. This trend can also be observed in other putative DMR region with lower, though still informative, RRBS coverage (Figure S5A). Methylation levels of the latter DMR regions are predicted remarkably well by RRBS despite the lower coverage. Investigation of the spatial organization of methylation patterns within these putative DMRs again shows high consistency in the distribution of methylation in ESCs and iPSCs (Figure 2G, Figure S5B). Notably, H1 WM data give rise to dramatically higher methylation levels across all of the regions. One possible explanation for this observation might be the generally higher cytosine methylation levels in the H1 WM data. Interestingly, nearly all of these mega-DMR regions were characterized by reduced CpA methylation levels compared to the surrounding DNA segments in ESC and iPSCs (Figure 2G and Figure S5B–S5D). Additionally, almost all potential DMRs exhibit a sharp drop in CpG density at the beginning of each region, followed by an increase towards the end (Figure S5E), while CpA density exhibited only small fluctuations. Taken together, these observations suggest that some iPSCs may deviate from a reference ESCs but in general they cannot solely be distinguished from ESCs based on non-CpG methylation patterns.

### CpA methylation levels correlate with *de novo* methyltransferase activity

**Figure 3:**
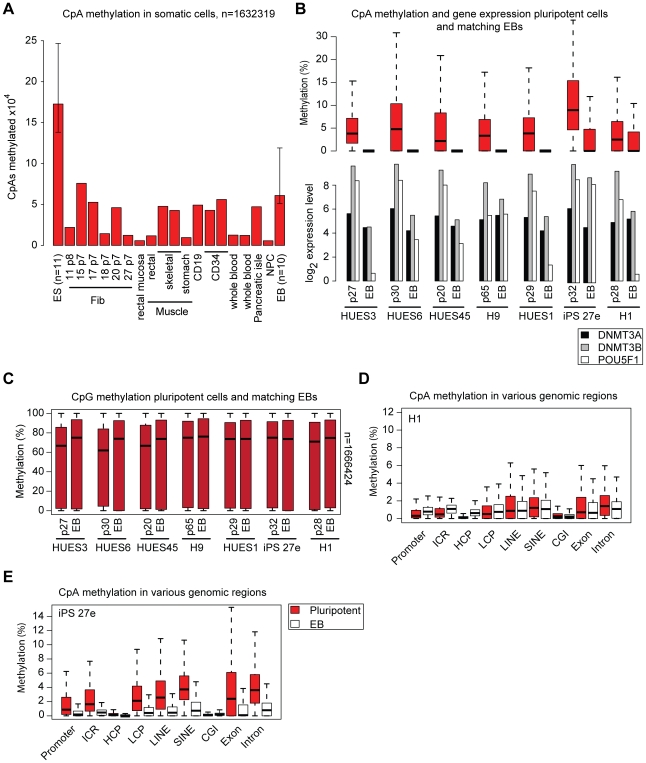
CpA methylation dynamics are closely linked to DNMT3 gene expression levels. (A) Number of CpAs (y-axis; value ×10^4^) methylated (≥5% methylation) in various somatic cell types and median number of methylated CpAs in EBs. Median number of methylated CpAs in a representative subset of ESCs (n = 11) is shown as reference. Whiskers indicate 25th and 75^th^ quartiles. (B) Distribution of CpA methylation levels in 7 pluripotent cell samples and matching 16 day EBs (top). Boxes are 25^th^ and 75^th^ quartiles, whiskers indicate most extreme data point less than 1.5 interquartile range from box and black bar represents the median. Below are normalized absolute log_2_ gene expression levels of DNMT3A, DNMT3B and OCT4 in the corresponding samples (measured using Affymetrix GeneChip HT HG-U133A microarrays; Table S1). Left sample in each pair corresponds to the undifferentiated state and right sample to the matching EB state. (C) Distribution of CpG methylation levels in 7 pluripotent cell lines and matching 16 day EBs. (D) CpA methylation levels of various genomic region classes in ESC line H1p38 and matching 16 EBs. (E) CpA methylation levels of various genomic region classes in iPSC line 27e matching day 16 EBs.

We next compared the presence of CpA methylation in pluripotent cells closely with those found in somatic cells. We therefore analyzed DNA methylation patterns in 10 somatic cell types (n = 18) representing all three germ layers (Figure 3A). As expected almost all cell types under consideration exhibit virtually no or very low levels of CpA methylation (nor any CpT or CpC (see also Figure 1G)).

To investigate the dynamics of CpA methylation during ESC/iPSC differentiation, we used day 16 EB samples derived from 10 of the pluripotent cell lines. As expected, CpA methylation levels decreased upon EB formation, albeit to variable degrees among lines (Figure 3A, 3B), While CpA methylation levels in some EBs drop to somatic levels, others still exhibit intermediate levels (Figure 3B). In contrast, global differences in CpG methylation were only marginal (Figure 3C). With the exception of H1 p38 and iPS27e, in all pluripotent cell - EB pairs the reduced CpA methylation was accompanied by the downregulation of pluripotency marker genes as well as the *de novo* DNA methyltransferases DNMT3A and DNMT3B (Figure 3B). Interestingly, EBs derived from our H1 p38 still showed higher levels of CpA methylation despite more than 6-fold down-regulation of OCT4. When we compared the distribution of methylation levels across multiple genomic regions it revealed no change of CpA methylation levels (Figure 3D). Notably, the *de novo* DNA methyltransferase DNMT3A, which is implicated in non-CpG methylation [2], remained expressed at ESC levels while DNMT3B expression experienced a 3-fold decrease (Figure 3B). In contrast, the iPS 27e cell line retained high levels of pluripotency gene expression upon EB differentiation and appears to be locked in the pluripotent state based on earlier studies [12], [17]. However, even though the majority of cells in the iPS27e EB population may still exhibit molecular pluripotency, a notable proportion may have experienced downregulation of the DNMTs (Figure 3B). These observations are in line with the reduced overall CpA methylation levels in the matching EBs (Figure 3B) as well as the unbiased reduction of CpA methylation in various genomic regions (Figure 3E). To investigate the relationship between non-CpG methylation and pluripotency associated genes more closely, we derived a linear model predicting the mean non-CpG methylation levels based on expression levels of selected marker genes in a representative subset of our samples (n = 37) for which matching DNA methylation and gene expression data were available (Table S1). Consistent with our analysis in EBs, this highly predictive linear model (r^2^ = 0.55, p = 0.0002) identifies DNMT3A and DNMT3B gene expression levels as being most associated with total sample CpA methylation levels. In contrast, pluripotency associated marker genes like OCT4, SOX2 or NANOG did not contribute significantly to predictive power. These results suggest that expression of pluripotency genes (molecular pluripotency) is not a necessary precondition for the presence of non-CpG methylation. Instead, these examples suggest an uncoupling of the core pluripotency network and non-CpG methylation levels and point towards DNMT3A and DNMT3B as a key effectors of the latter.

### Depletion of DNMT3A results in global reduction of non-CpG methylation

**Figure 4:**
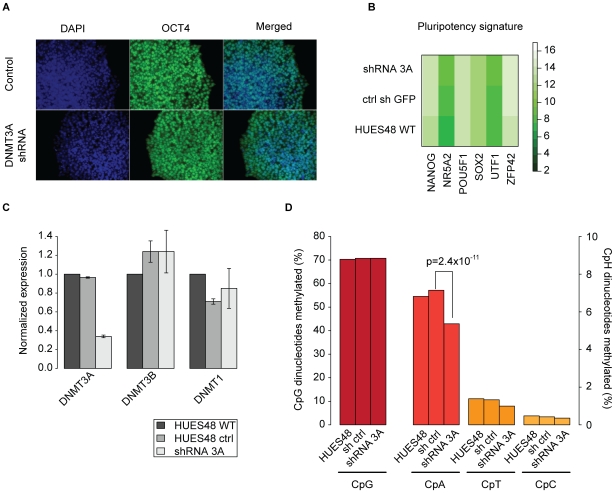
Knockdown of DNMT3A in hESCs causes global reduction of non-CpG methylation. (A) OCT4 immunostaining of representative ES cell line HUES48 infected with a control shRNA and a shRNAs against DNMT3A. (B) Expression of various pluripotency associated genes in HUES48 infected with shRNAs against DNMT3A and controls as assessed by the Nanostring nCounter. (C) qRT-PCR of DNMT3A in HUES48 WT, HUES48 infected with shRNAs against DNMT3A and control shRNA against GFP. Expression values are normalized to β-Actin levels. (D) Percentage of methylated (≥10%) cytosine dinucleotides in HUES48 treated with shRNAs against DNMT3A and control samples. P-value was determined using Wilcoxon-rank test.

In order to experimentally support these bioinformatics models we decided to stably knockdown DNMT3A in hESC line HUES48 using shRNAs. Infected HUES48 ESCs showed an undifferentiated morphology and remained molecularly pluripotent as confirmed by OCT4 staining (Figure 4A) and gene expression profiling (Figure 4B). Next, we assessed the knockdown efficiency for DNMT3A and found an approximate reduction of 70% by quantitative real-time PCR (Figure 4C, Figure S6A). We observe mild differences for the other DNMTs between the original, uninfected HUES48 and the infected cell lines (shRNA control and shRNA DNMT3A), which are likely due to clonal expansion post infection. To evaluate the impact of DNMT3A knockdown on the abundance of non-CpG methylation, we analyzed HUES48 WT, HUES48 infected with control shRNA and HUES48 infected with shRNA against DNMT3A using RRBS. As expected we do not observe notable changes in global CpG methylation levels. However, we find a 28% reduction in the number of methylated CpAs compared to the control sample (p-value = 2.497 10^−11^ Wilcoxon-rank test, Figure 4D). This reduction is also reflected in the number of methylated CpTs and CpCs. It should be noted that the reduction of non-CpG levels might become even more dramatic with increased passage numbers given our incomplete knockdown of DNMT3A. To confirm our findings, we repeated the knockdown in hES cell line H1. Consistent with the results for HUES48, we observed a 33% reduction in the number of methylated CpAs while CpG methylation levels were not affected (Figure S6B). Additionally, we utilized a shRNA against DNMT3B to probe its role in non-CpG methylation (Figure S6B,C) and observed a 82% reduction in the number of methylated CpAs while CpG methylation was again not affected (Figure S6B). These results clearly show that both *de novo* methyltransferases are significant sources of non-CpG methylation in hESCs.

### CpA methylation is spatially correlated with CpG methylation

**Figure 5:**
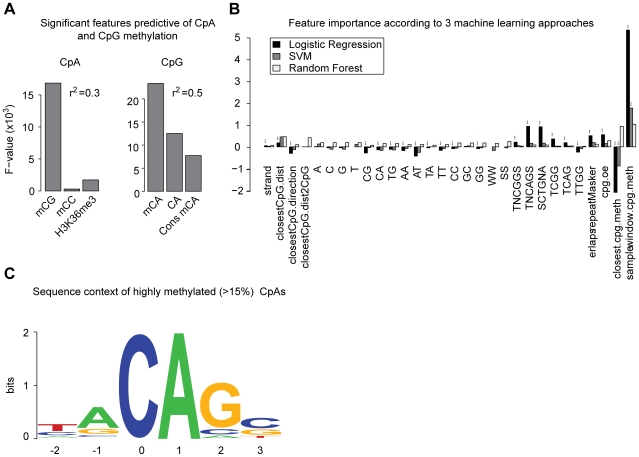
Genomic context and attributes of CpA methylation. (A) Significant and most influential features predictive for CpA methylation in a linear model based on a 1 kb tiling of the human genome covered by RRBS (n = 32300 tiles). The linear model included classical sequence features (but excluding CpG density) as well as methylation of CpG, CpT, CpC, H3K36me3 methylation and conservation of CpA methylation state. F-statistics reported for 9 and 32291 degrees of freedom. (B) Feature importance for prediction of CpA methylation according to three machine learning approaches. Depicted are logistic regression and linear SVM weights (black and dark grey, respectively) as well as feature Mean Decrease in Gini Index (MDG, light grey) according to random forests (rescaled such that the largest MDG corresponds to 1). Significant features characterized by a p-value <0.05 for logistic regression or a z-score >1.96 for linear SVM are marked (***). A detailed description of features is given in Table S2. (C) Sequence context of consistently highly methylated (mean ≥15%) CpAs (n = 5551) over all ES cell lines n = 30.

Our data confirmed the presence of non-CpG methylation in pluripotent cells, however little is known about the relevance of this modification and whether it plays any regulatory role. This is difficult to experimentally test, but we wanted to computationally assess if CpA methylation might constitute an independent regulatory mechanism. Towards this end, we employed a linear regression model for the prediction of the CpA methylation state of a 1 kb tiling of all regions covered by RRBS. Out of eight features (see Materials and Methods) tested for each region, CpC methylation, CpG methylation and the presence of a histone modification proved to be significant and predictive with an overall variance explained of r^2^ = 0.3 (Figure 5A, left). Out of these three features, the CpG methylation state is by far the most predictive (Figure 5A, left), as assessed by ANOVA. The tri-methylation state of lysine 36 in histone 3 (H3K36me3) which has been shown to be enriched in gene bodies of transcribed genes [20] ranked second. On the background of our findings on DNMT3A's role in establishing non-CpG methylation patterns and recent reports showing recruitment of murine Dnmt3a through its interaction with H3K36me3 through the PWWP domain [21] these findings might provide an explanation for the previously reported correlation of non-CpG methylation levels in gene bodies and gene expression [9]. However, given the moderate predictive performance of the linear model, the high inter-sample variability of CpA methylation and the fact that CpG methylation is widely present without CpA methylation, we next reversed the question and tried to predict CpG methylation based on CpA methylation levels and sequence features. This second linear model proved to be of superior predictive performance with r^2^ = 0.5 (Figure 5A, right). Interestingly, CpA methylation levels turned out to be the most influential predictor of CpG methylation, followed by CpA density and conservation of CpA methylation levels across multiple samples (Figure 5A, right). The observation that CpA methylation is a strong predictor of CpG methylation (r^2^ = 0.32) suggests a strong link of CpG and CpA methylation. In combination with the dramatically lower predictive performance of CpG methylation for CpA methylation, these findings point towards a possible dependence of CpA methylation on CpG methylation.

We next applied a comprehensive classification approach in order to characterize traits determining CpA methylation and computationally analyzed the genomic and epigenomic context in which CpA methylation occurs. We employed multiple statistical learning procedures known for their predictive potential and interpretability, and assessed the resulting models using 10-fold cross-validation. Analysis of the Area Under ROC Curves (AUC) revealed high predictive power and information content of the inferred models (Figure S7D, mean AUC = 0.78 (logistic regression and linear kernel SVMs) and 0.83 (random forest). The methylation state of CpGs in close vicinity, the distance to the closest CpG as well as the preferential flanking sequence of DNMT3A [6], [22] appear among the most predictive features across methods (Figure 5B). In particular, the sequence context of highly methylated CpAs (>15%) that are also more conserved between samples is reminiscent of the weak sequence preference of DNMT3A (Figure 5C) [6], [22]. Interestingly, this particular sequence motif has recently been reported to be enriched around highly methylated CpAs in whole genome bisulfite sequencing data located at splicing sites [23]. In summary, our findings point toward a close link of CpG and non-CpG methylation in terms of their spatial distribution.

## Discussion

RRBS offers the opportunity to investigate methylation states of a representative fraction of cytosines in the genome across large numbers of samples. In this study, we utilized a large data set comprising pluripotent and differentiated human cell types to investigate patterns of non-CpG methylation. Our analysis allowed us to assess the intrinsic variability in different classes of cytosine methylation and identify possible effectors.

Overall our study finds non-CpG methylation to be a rare and highly variable modification. We confirmed previous reports that non-CpG methylation levels are high in pluripotent cells and that somatic cell types exhibit low levels of non-CpG methylation [2], [7], [9]. We further confirm that non-CpG methylation patterns are generally reestablished upon transcription factor induced reprogramming [11], but find no consistent differences between ESCs and iPSCs in terms of non-CpG methylation when comparing more than 30 pluripotent cell lines. We also show that in general non-CpG methylation is lost relatively early during EB formation. However, our results indicate that non-CpG methylation might not be attributed directly to the pluripotent state but rather linked to the *de novo* methyltransferases DNMT3A and DNMT3B as key effectors. We further support this hypothesis by knockdown of DNTM3A and DNMT3B in hESCs, which results in a global reduction of CpA methylation levels while molecular pluripotency was unaffected. Both DNMT3A and DNMT3B are highly expressed in human ESCs and iPSCs and downregulated during normal differentiation. Previous murine data showed that early passage 3a/3b double knockout mouse ES cells, which lack non-CpG methylation, are still capable of differentiation using various assays and retain their self-renewing capacity [24]. In agreement with these experimental results, our findings suggest that non-CpG methylation is dispensable for pluripotency.

We also find that CpA methylation does not appear to be generally decoupled from CpG methylation and genomic determinants thereof. This is consistent with a recent report in murine early embryos that not only showed non-CpG methylation within or near regions of high CpG methylation at DMRs, but also its absence from unmethylated regions such as the associated paternal allele [25]. Interestingly, we observe high correlation of CpA methylation with the presence of methylated CpGs in close vicinity. Considering the current evidence, it seems likely that the majority of the observed CpA methylation is of stochastic nature due to unspecific activity of DNMT3A and 3B. However, a small fraction of the highly methylated non-CpGs exhibits high conservation of methylation levels across samples and might be of functional relevance. One specific function might be in the transient silencing of low CpG density repeats during genome wide remodeling processes (Figure S7E). It would be interesting to test this hypothesis in more detail to uncover functional roles of non-CpG methylation or to characterize non-GC methylation as a consequence of unspecific DNMT3 activity. The global presence of non-CpG methylation indicates that the *de novo* methyltransferases operate in a widespread manner in addition to a contained recruitment to specific loci. This observation is consistent with previous reports of gradual global loss of DNA methylation upon long-term culture of Dnmt3a/3b double knockout mouse ES cells [24] and the suggested additional role of DNMT3A/3B in the correction of errors made by DNMT1 [26].

In summary our data provides new insights into the genomic distribution of DNA methylation in a large sample set of human pluripotent and differentiated cells. Better understanding of the non-CpG methylation landscape helps clarify recently raised questions about its role in human pluripotency and will provide a useful basis for future experimental validations.

## Materials and Methods

### Cell lines and samples

A total of 20 human ES cell lines, 12 human iPS cell lines, as well as 10 distinct somatic cell types were investigated in this study (Table S1). The ES cell lines were originally obtained through the Human Embryonic Stem Cell Facility of Harvard University (17 ES cell lines) and from the WiCell Research Institute's WISC Bank (3 ES cell lines) [12]. The iPS cell lines were derived by retroviral transduction of OCT4, SOX2, and KLF4 in dermal fibroblasts [17]. All pluripotent cell lines have been characterized by conventional methods [27], [28] and were grown under standardized conditions as described before [12]. Embryoid bodies (EBs) and fibroblast samples were also taken from the previous study [12] (Table S1). The material for the rectal mucosa, rectal smooth muscle, skeletal muscle and stomach smooth muscle samples was obtained from MGH Pathology under the NIH Roadmap Epigenomics Program and processed by the Broad's Reference Epigenome Mapping Center (REMC). The H9 derived NPC sample was obtained from ArunA biomedical under the NIH Roadmap Epigenomics Program and processed by the REMC. Human blood CD19 and CD34 samples were obtained from Shelly Heimfeld's lab as part of the REMC. Pancreatic islet samples were retrieved from the islet donor network and supplied by Stuart Schreibers group at the Broad. All data sets and additional (matched) chromatin maps that are generated as part of the NIH Roadmap project and not included in the manuscript are publically available (http://www.roadmapepigenomics.org/).

### DNA methylation mapping and data processing

RRBS was performed according to a previously published protocol [29], incorporating some optimizations for small cell numbers [30]. Raw sequencing reads were aligned to the Msp-I digested and *in silico* size selected human genome using MAQ's bisulfite alignment mode [31]. DNA methylation calling was performed using custom software [30]. For all covered cytosines DNA methylation levels of individual cytosine dinucleotides were assessed by the fraction of reads exhibiting an unconverted cytosine over total number of reads.

For comparison of RRBS and whole methylome (WM) data [9], [10], published raw data was retrieved and processed by a custom software [19]. For WM and RRBS, cytosines were filtered for 5x minimum read coverage. Subsequently, methylated non-CpGs in WM and RRBS data were defined as those exhibiting a methylation ratio above 5%. For CpGs, the cutoff was set to a 30% methylation ratio. Based on these definitions, the comparison of methylated CpGs and non-CpGs in Figure 1D and Figure S1B was performed on the set of cytosine dinucleotides fulfilling the minimum coverage criteria in WM and RRBS data. Bisulfite conversion rate was assessed through the global mean levels of CpC methylation and methylation levels in a subset of promoters overlapping with CG islands for each individual sample (Table S1).

Comparing different coverage cutoffs, we find that CpA methylation distributions differ compared to the distribution computed only on those CpAs with more than 50x coverage. Based on this analysis we chose a coverage cutoff of 5x in 80% in pluripotent and differentiated cells yielding 1.7 million in pluripotent and 1.6 million CpAs in differentiated cells respectively. While resulting in a reasonable number of CpAs for analysis, this cutoff is associated with a slight deviation from the >50x coverage regime. However, due to sequencing and processing bias the 50x cutoff distribution is not representative of the true distribution either. Due to the large number of sampled CpAs the confounding effect of the 5x coverage threshold is likely to be attenuated. In order to investigate the distribution of methylated cytosines and avoid domination of the population by the unmethylated cytosines, we imposed a minimal methylation threshold on cytosine dinucleotides (relevant for Figure 1F, Figure 2D, Figure 3B and 3C, and Figure S3C–S3E). This partitioning is particularly important when investigating non-CpG methylation since the vast majority of the non-CpGs doesn't show any evidence for methylation. The threshold was set to 0.1% median methylation for CpG as well as non-CpG dinucleotides over all pluripotent samples. Based on CpAs and CpGs fulfilling these criteria, we computed the coefficient of variation (standard deviation divided by mean) for the methylation level of individual dinucleotides over all ES cell samples (n = 30) in Figure 2C.

To analyze the sequence context of highly methylated non-CpG dinucleotides with ≥10x coverage in ≥80% of all ES cell samples, we identified CpAs showing more than 15% mean methylation over all ES cells lines (n = 30), Figure 5C. Subsequently, we computed base frequencies around the 5551 identified CpAs and created Figure 5C using WebLogo [32].

Infinium analysis was performed by the Genetic Analysis Platform at the Broad Institute. A total of 1 µg of genomic DNA per sample was bisulfite-treated according the manufacturers protocol and hybridized onto Human InfiniumMethylation 450 bead arrays (Illumina). Raw data was processed using the Illumina GenomeStudio software. Probes with a detection p-value >0.05 were discarded. For comparing RRBS assayed methylation to the array data, the mean RRBS methylation level in a region of 100 bp up and downstream of the Infinium probe was taken into account.

### Gene expression data

Microarray gene expression data were taken from our previously published data set [12] and normalized to the mean expression levels in ES cells (n = 20).

### Genomic features

Cytosine methylation levels were calculated for distinct classes of genomic features: Promoters were defined as a −5 kb to +1 kb sequence window surrounding the annotated transcription start site of Ensembl-annoted genes [33]. CG islands were defined according to CAP-seq results reported in [34]. Promoters overlapping with a CpG island were defined as CG island promoters, others as Non-CG island promoters, imprinting control regions were manually curated based on published results, intron and exon regions were downloaded from the USCS (http://genome.ucsc.edu/) for all ensembl genes. SINE and LINE element annotation was taken from the Repeat Masker/Repbase information provided by the USCS genome browser.

### Non-CpG DMRs

Regions that were reported to be consistently hypomethylated in 5 iPS cells compared to the H1 human ES cell line were taken from [11] and investigated in ES (n = 20) and iPS cells (n = 12) utilizing our RRBS data set. Each region was tiled into 1 kb intervals and the methylation state of each segment was determined based on all CpAs exhibiting a minimum read coverage of 5x in all samples in order to insure comparability. Subsequently, only those regions with at least 0.5% of all CpAs covered were retained and analyzed (Figure S5A).

For these regions, the median methylation was calculated based on the 1 kb region tiling and depicted in Figure 2F and Figure S5A. In Figure 2G the CpA methyalation state of each 1 kb window over a selected, 2 Mb DMR on chromosome 22 as well as its vicinity is shown. Trend lines were added based on spline smoothing. The average CpG content profile for each of the 22 DMRs reported in [11] was computed (Figure S5E) by dividing each region into 30 equally long sequence intervals and calculating the CpG content of each interval. Subsequently, matching intervals from all regions were averaged and plotted. In addition, the average CpG content for 30 kb up- and downstream of each region was averaged in 1 kb intervals for all regions and plotted as well.

### Analysis of CpA methylation in differentiated cells

For all differentiated cell samples we determined a consensus set of 1.6 million CpAs with a minimum coverage of 5-fold in 70% of all samples within this class. In addition, these CpAs were also covered by at least 5x in a set of 11 reference ES cell samples. All CpAs with a methylation level of ≥5% were counted as methylation events and are depicted in Figure 3A.

### Linear model and ANOVA analysis

In order to determine the relationship between non-CpG methylation, pluripotency and DNMT gene expression levels, we employed a linear model trained on 32 pluripotent and 10 EB samples for which we had DNA methylation and microarray data. Using as the response variable the median methylation level of 1.5 million CpA's consistently covered in all samples we trained a linear model on the gene expression levels of DNMT3A/B, DNMT1, OCT4, SOX2 and NANOG. Following this analysis we performed ANOVA utilizing the statistical programming language R (http://www.r-project.org/) with built in functions.

A second linear regression model was employed to determine the potential of CpA methylation to constitute an independent regulatory mechanism. Using the linear model, we simultaneously controlled for other classical predictive genomic features such as: A,C,G,T content, repeat content and CpA content as well CpA-CpG ratio. We specifically excluded CpG density related variables from our prediction approach to avoid dominance of this highly predictive feature of DNA methylation. In addition, we computed a methylation conservation score over 10 representative ES cell lines for each individual CpA. For this, we determined the coverage weighted mean and standard deviation over all pluripotent samples (n = 42). To balance high and low coverage contributions, we limited the maximum coverage contribution of an individual CpA to 25. The conservation score was then defined as the ratio of the weighted standard deviation and the weighted mean. Subsequently, we investigated the predictive power of these genomic features to infer CpA methylation status on 32,000 1 kb regions with a minimum of two CpA dinucleotides per region consistently covered more than 5x between all 10 samples. This analysis was again followed by ANOVA. Subsequently we used the same approach to predict CpT and CpG methylation based on the same features but now incorporating CpA methylation levels as predictor variable.

### Determining traits of CpA methylation using a classification approach

For CpA dinucleotides with coverage of at least 15 reads in all of 5 representative samples we determined 30 features (see Table S2). Sequence features were computed for a window of 10 basepairs upstream and downstream of the respective CpA. A threshold of 5% was applied to group the data into methylated and unmethylated dinucleotides respectively. In order to remove the data set's bias towards unmethylated CpAs, unmethylated datapoints were randomly sampled to match the number of methylated ones. This resulted in data sets of sizes 37628, 77474, 44010, 104512 for the “hES H1 p25”, “hES H1 p34”, “hES H9 p58”, “hiPS 15bp33” and “hiPS27e p32” samples respectively.

Subsequently, three classification methods were used: (1) logistic regression, (2) support vector machines employing a linear kernel, and (3) random forests with the number of trees set to 500 (summarized in [35]). All three methods were applied to each of the 10-fold cross-validation subsets and mean AUCs were computed among the 10 subsets.

Feature contributions were calculated from models derived from the full training set. For logistic regression and linear SVMs they were assessed as the variable coefficients. Z-scores were used to infer statistical significance. For random forests, the mean decrease in Gini Index served as importance measure.

The R statistical programming language (version 2.12) with the e1071, randomForest and ROCR packages was used to conduct the analysis. The genome version used was UCSC hg18 from the BSgenome package of Bioconductor.

Data processing was performed by custom Python (http://python.org/) and R scripts.

### Knockdown of DNMT3A and DNMT3B

DNMT3A was stably knocked down in hESC HUES48 using shRNAs from The RNAinterference consortium (TRC; http://www.broadinstitute.org/rnai/trc).

shRNA against DNMT3A (TRCN0000035755; target: CCGGCTCTTCTTTGAGTTCTA)

shRNA against DNMT3B (TRCN0000035684; target: GCCTCAAGACAAATTGCTATA)

control: anti GFP (TRCN0000072199; target: TGACCCTGAAGTTCATCTGCA).

HUES48 and H1 were infected and selected for 10 days with puromycin. Cells were passaged 5 times before material for qRT-PCR, Nanostring and RRBS profiling was collected.

### Quantitative RT–PCR

RNA was extracted using RNeasy kit (QIAGEN). The cDNA was synthesized from 2 µg of total RNA using RevertAid™ First Strand cDNA Synthesis Kit (Fermentas). The primers used for quantification were as follows:

DNMT3A forward (F): 5′-GCTCTTTGAGAATGTGGTGG-3′, and reverse (R): 5′-CTTTGCTGAACTTGGCTATCC-3′; DNMT3B F, 5′-GAGTCCATTGCTGTTGGAACCG-3′, and R, 5′-ATGTCCCTCTTGTCGCCAACCT-3′, DNMT1 F1

5′-GGGAAGACCTACTTCTACCAG-3′ and R1 5′-ACAGCTTGATGTTGAACGTG-3′, DNMT1 F2 5′ AGTTTGTGAGCAACATAACCAG-3′ and R2

5′-CACTCATGTCCTTACAGATGTG-3′, β-ACTIN F

5′-TTTGAGACCTTCAACACCCCAGCC-3′ and R 5′ AATGTCACGCACGATTTCCCGC-3′.

Gene expression levels were measured using an ABI 96 well Step 1 Plus RT PCR System and SYBR Green PCR Reagents (Applied Biosystems).

### Nanostring profiling

RNA was extracted using the RNeasy kit (QIAGEN). Subsequently, 500 ng of RNA was profiled on the NanoString nCounter system according to manufacturer's instructions. A custom nCounter codeset was used which covers 556 genes; subsequent data analysis was performed according to a previously published protocol [12].

## Supporting Information

Figure S1 Characteristics of non-CpG methylation in pluripotent cells. (A) Percentage of key genomic features covered by RRBS. (B) Venn diagrams show the overlap of methylated CpGs (top) as well as non-CpGs (bottom) in HUES64 (p19 and p36) that exhibit above threshold (≥10% and ≥5% methylation) methylation in whole methylome and RRBS data of the same sample. Only those dinucleotides were considered that were covered in both data sets simultaneously by at least 5 reads in order to estimate the conservation of methylation events. Numbers below venn diagram indicate overlap of both dinucleotide sets. (C) Distribution of CpA dinucleotide coverage in RRBS data over all pluripotent samples. (D) Spatial distribution of CpG (black) and CpA (red) methylation levels over various genomic features for RRBS (dashed line) and whole methylome data (HUES64).
(TIF)

Figure S2 Locus-specific bisulfite sequencing confirms RRBS based CpG and non-CpG methylation state of selected genomic regions. (A) Methylation state of CpAs located in the NPPA gene on chromosome 1 according to RRBS (top) and locus-specific bisulfite sequencing (bottom). Shown on top are the locations of CpGs (dark red) and CpAs (red) as well as the number of methylated/total reads covering a particular position. Shown in the middle are the locations of MspI (black rectangle) sites as well as the location and extend of sequencing reads. Depicted below are bisulfite sequencing results indicating the methylation state of CpGs (circles) and CpAs (rectangles). Methylation data are shown for individual clones with solid black forms corresponding to methylated cytosines. (B) Methylation state of CpAs located in the Krt17 gene on chromosome 17 according to RRBS (top) and locus-specific bisulfite sequencing (bottom). (C) Methylation state of CpAs located in the PTP4A3 gene on chromosome 8 according to RRBS (top) and locus-specific bisulfite sequencing (bottom).
(TIF)

Figure S3 Distribution of non-CpG and CpG methylation in pluripotent cells. (A) Pearson correlation coefficients of individual CpC dinucleotide methylation levels in six replicates of H1. (B) Overall chromosomal distribution of CpA methylation levels in H1p25. (C) Boxplots show CpA methylation levels for all ESC samples on a set of 290,462. CpAs with coverage of at least 5x in more than 80% of all ESC samples and median methylation of ≥0.1%. Boxes are 25^th^ and 75^th^ quartiles, whiskers indicate most extreme data point less than 1.5 interquartile range from box and black bar represents the median. (D) Boxplots show CpG methylation levels for all ESC samples on a set of 2 million CpGs with coverage of at least 5x in more than 80% of all ES cell samples. Boxplots are defined as in C. (E) Distribution of CpG methylation levels in 12 iPSC lines and 7 ESC lines as a reference. Boxplots are based on ∼1.4 million CpGs that show more than 0.1% median methylation levels of 0.1% in the representative ES cell lines (n = 7). Blue boxes indicate samples with the two highest CpA methylation levels relative to the average over all pluripotent cell lines. Boxplots are defined as in C. (F) CpG methylation levels in different genomic region classes in ESC line H1 (p25, p30, p34, p37 and p38; n = 6, white) and iPSC lines 11a, 27e showing high overall CpA methylation levels (n = 2, blue). Genomic features are defined in the Materials and Methods.
(TIF)

Figure S4 Analysis of methylation in pluripotent cells using the Illumina Infinium 450K array. (A) Distribution of CpG methylation levels in ESCs and iPSCs. Boxes are 25^th^ and 75^th^ quartiles, whiskers indicate most extreme data point less than 1.5 interquartile range from box and black bar represents the median. (B) Distribution of CpA methylation levels in ESCs and iPSCs. Boxes are defined as in A. (C) The venn diagrams show the CpG and CpA dinucleotides covered by RRBS and Infinium 450 K array based on a 40–260 bp size selection. (D) RRBS read coverage distribution for matching samples profiled by Infinium 450 K and RRBS as well as HUES64 WM data. Boxes are defined as in A. (E) Heatmap showing pearson correlation coefficients of CpG methylation levels for matching pluripotent samples based on regions harboring CpGs covered by both RRBS and Infinium 450 K. (F) Heatmap showing pearson correlation coefficients of CpA methylation levels for matching pluripotent samples based on regions harboring CpAs covered by both RRBS and Infinium 450 K. (G) CpG methylation levels of various genomic features according to the Infinium 450 K array. (H) CpA methylation levels of various genomic features according to the Infinium 450 K array.
(TIF)

Figure S5 ESCs and iPSCs show no consistent differences in putative DMR regions. (A) CpA methylation levels for 21 putative DMRs reported by Lister et al. 2011 using 20 ESCs (RRBS, boxplots) as a reference, HUES64 WM as well as previously published H1 and iPS ADS WM data [9], [11]. A. (B–D) CpA methylation profile of selected DMRs (framed by black lines) reported by Lister et al. 2011 based on a 1 kb tiling. The CpA methylation levels based on RRBS are shown for the median of all ESCs (n = 20) and all iPSCs (n = 12) as well as for H1p25 WM, iPS ADS WM and HUES64 WM. Regions were selected based on sufficient RRBS coverage (see Materials and Methods). (E) CpG density averaged over all putative DMRs based on a 100 bin tiling for each region. Black bars indicate start and end of putative DMRs.
(TIF)

Figure S6 Knockdown of DNMT3A and DNMT3B. (A) Location of PCR primers and shRNA target region in the DNMT3A and DNMT3B gene. (B) Percentage of methylated (≥10%) cytosine dinucleotides in H1 treated with shRNAs against DNMT3A, DNMT3B and control samples. (C) qRT-PCR of DNMT3A in H1 WT, H1 infected with shRNAs against DNMT3A, DNMT3B and control shRNA against GFP. Expression values are normalized to β-Actin levels.
(TIF)

Figure S7 The bona fide DNMT3A target region upstream of H19 shows high CpA methylation levels. (A) Spatial distribution of CpA methylation levels for two ESC lines upstream of the H19 locus. (B) Number of CpAs associated as a function of CpG density based on a genome wide 1 kb tiling. (C) Feature ranking for linear model predicting CpT methylation levels based on ANOVA Only the three most significant features are shown (p-value≤0.000187). Same feature combination as for Figure 5A, 5B was used (Materials and Methods). F-statistics was computed on 9 and 32291 degrees of freedom. (D) Repeat class LTR43 showing the highest CpA methylation levels observed while exhibiting extremely low CpG density. Mean CpG (top) and CpA (bottom) methylation levels obtained from aligning RRBS reads to a pseudogenome consisting prototypic repeat elements (RepBase Update) [19] are shown for 8 representative samples. Coloring corresponds to methylation level (dark red: unmethylated, light red: methylated). Labels in boxes represent percentage of methylation and read covereage. To the right, mean methylation levels across the 8 samples are given along with their odds ratio. (E) ROC curves of three machine learning methods to classify CpA methylation levels. mean AUCs across 10-fold cross-validation was 0.78 for logistic regression and linear support vector machine prediction and 0.83 for random forests. Error bars represent standard deviations. log.reg: logistic regression, smv.lin: linear SVM, rf: random forest.
(TIF)

Table S1 Quality measures and summary information for all individual samples included in this study. Informative reads are defined as successfully aligned reads that passed all quality controls and contained at least one of the indicated cytosine dinucleotides. UniqueSeqMotifCount specifies the number number of unique cytosine dinucleotide patterns observed in the genome based on the informative reads. Global methylation mean gives the mean methylation level over all cytosine dinucleotides covered for each sample. For the conversion rate, two estimates are given: one based on the global CpC methylation level and one computed through the average methylation level of 30 high CpG density promoters in each sample.
(XLSX)

Table S2 Extended features used to train three machine-learning methods for the prediction of the methylation state of individual CpAs.
(XLSX)
