# Supplementary figures and images for: Genomic Distribution and Inter-Sample Variation of Non-CpG Methylation across Human Cell Types
Source: PLoS Genet. 2011 Dec 8;7(12):e1002389. doi: 10.1371/journal.pgen.1002389 (PMC3234221; doi:10.1371/journal.pgen.1002389)

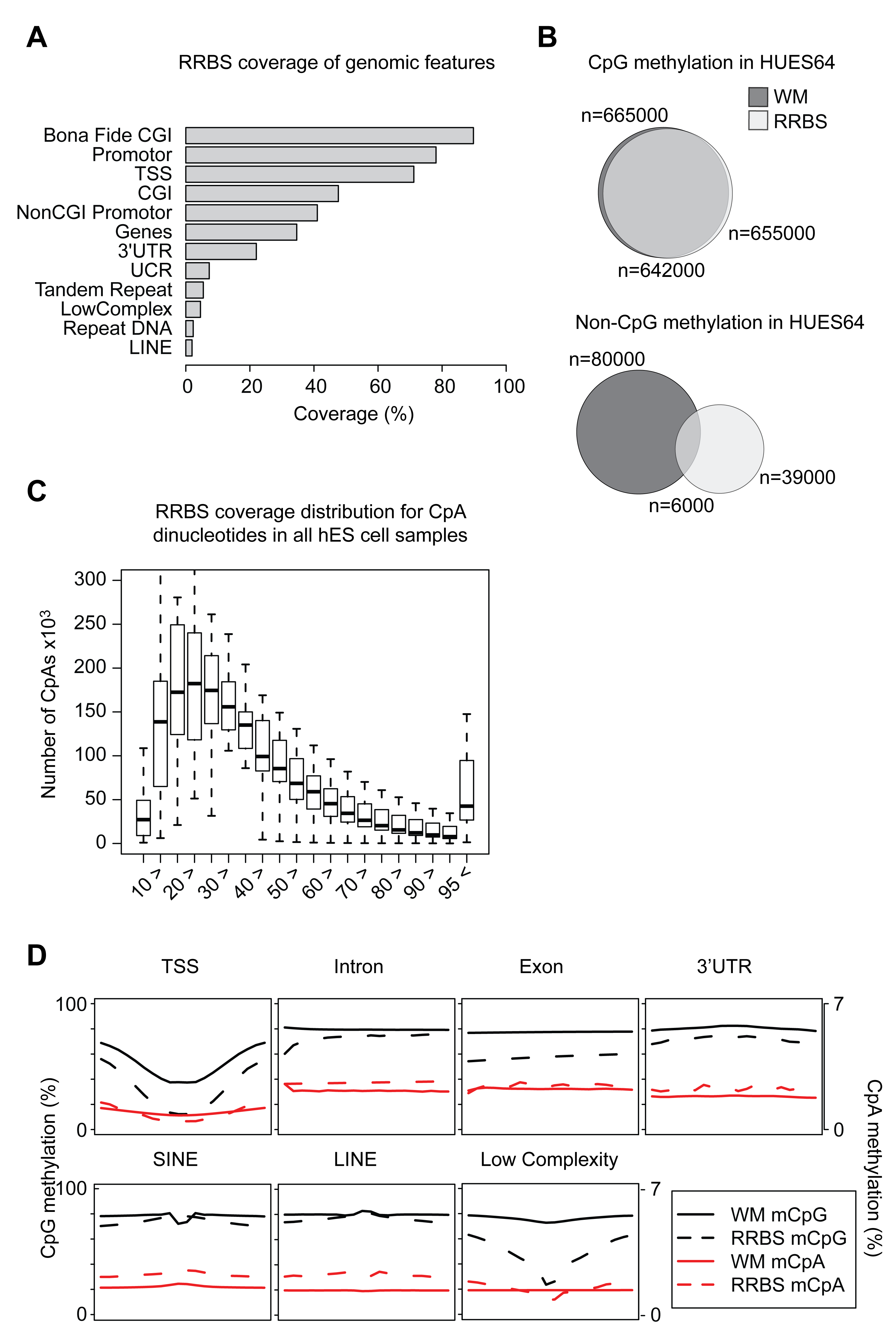

Supplement: Figure S1 — Characteristics of non-CpG methylation in pluripotent cells. (A) Percentage of key genomic features covered by RRBS. (B) Venn diagrams show the overlap of methylated CpGs (top) as well as non-CpGs (bottom) in HUES64 (p19 and p36) that exhibit above threshold (≥10% and ≥5% methylation) methylation in whole methylome and RRBS data of the same sample. Only those dinucleotides were considered that were covered in both data sets simultaneously by at least 5 reads in order to estimate the conservation of methylation events. Numbers below venn diagram indicate overlap of both dinucleotide sets. (C) Distribution of CpA dinucleotide coverage in RRBS data over all pluripotent samples. (D) Spatial distribution of CpG (black) and CpA (red) methylation levels over various genomic features for RRBS (dashed line) and whole methylome data (HUES64). (TIF) [file pgen.1002389.s001.tif]

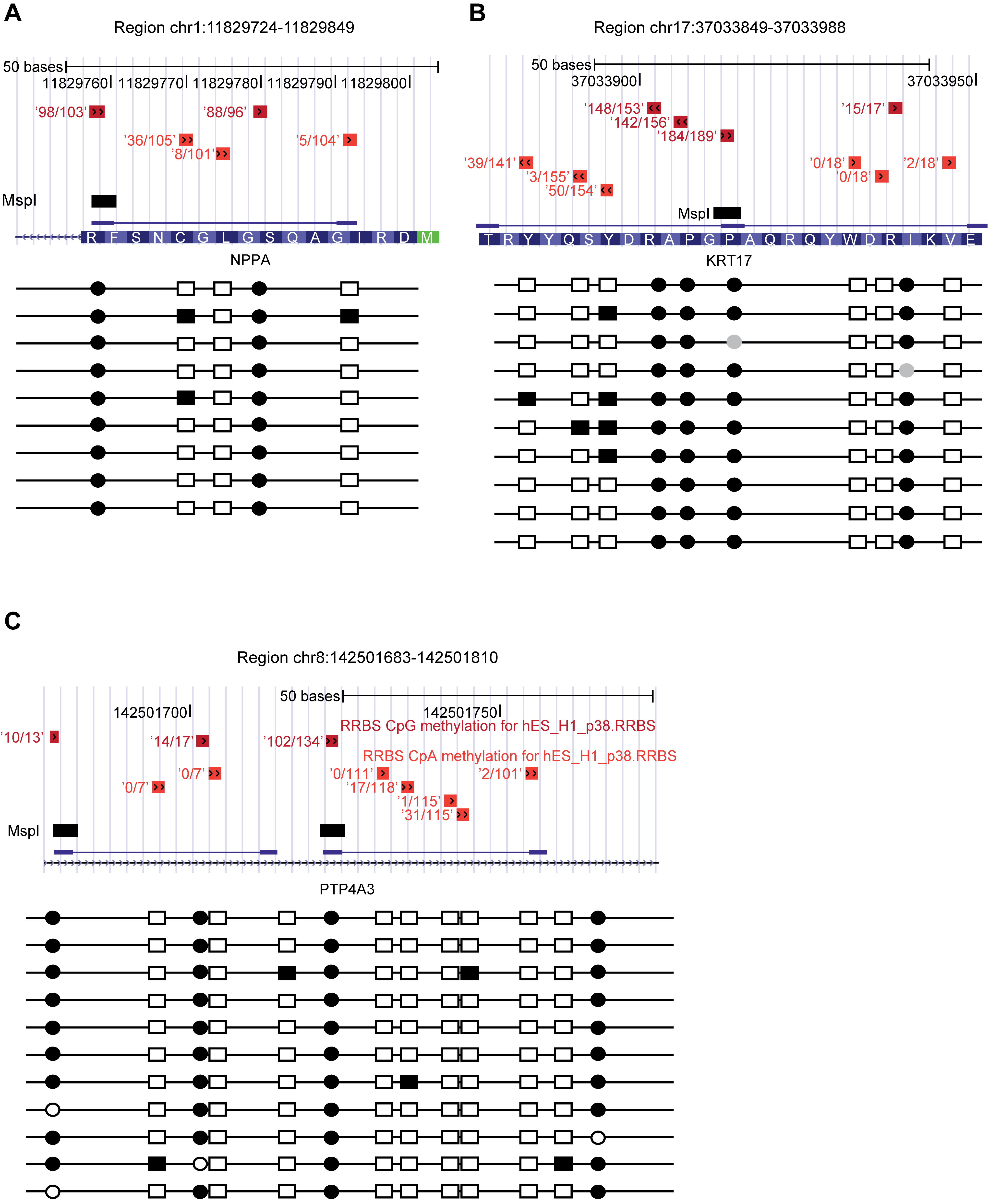

Supplement: Figure S2 — Locus-specific bisulfite sequencing confirms RRBS based CpG and non-CpG methylation state of selected genomic regions. (A) Methylation state of CpAs located in the NPPA gene on chromosome 1 according to RRBS (top) and locus-specific bisulfite sequencing (bottom). Shown on top are the locations of CpGs (dark red) and CpAs (red) as well as the number of methylated/total reads covering a particular position. Shown in the middle are the locations of MspI (black rectangle) sites as well as the location and extend of sequencing reads. Depicted below are bisulfite sequencing results indicating the methylation state of CpGs (circles) and CpAs (rectangles). Methylation data are shown for individual clones with solid black forms corresponding to methylated cytosines. (B) Methylation state of CpAs located in the Krt17 gene on chromosome 17 according to RRBS (top) and locus-specific bisulfite sequencing (bottom). (C) Methylation state of CpAs located in the PTP4A3 gene on chromosome 8 according to RRBS (top) and locus-specific bisulfite sequencing (bottom). (TIF) [file pgen.1002389.s002.tif]

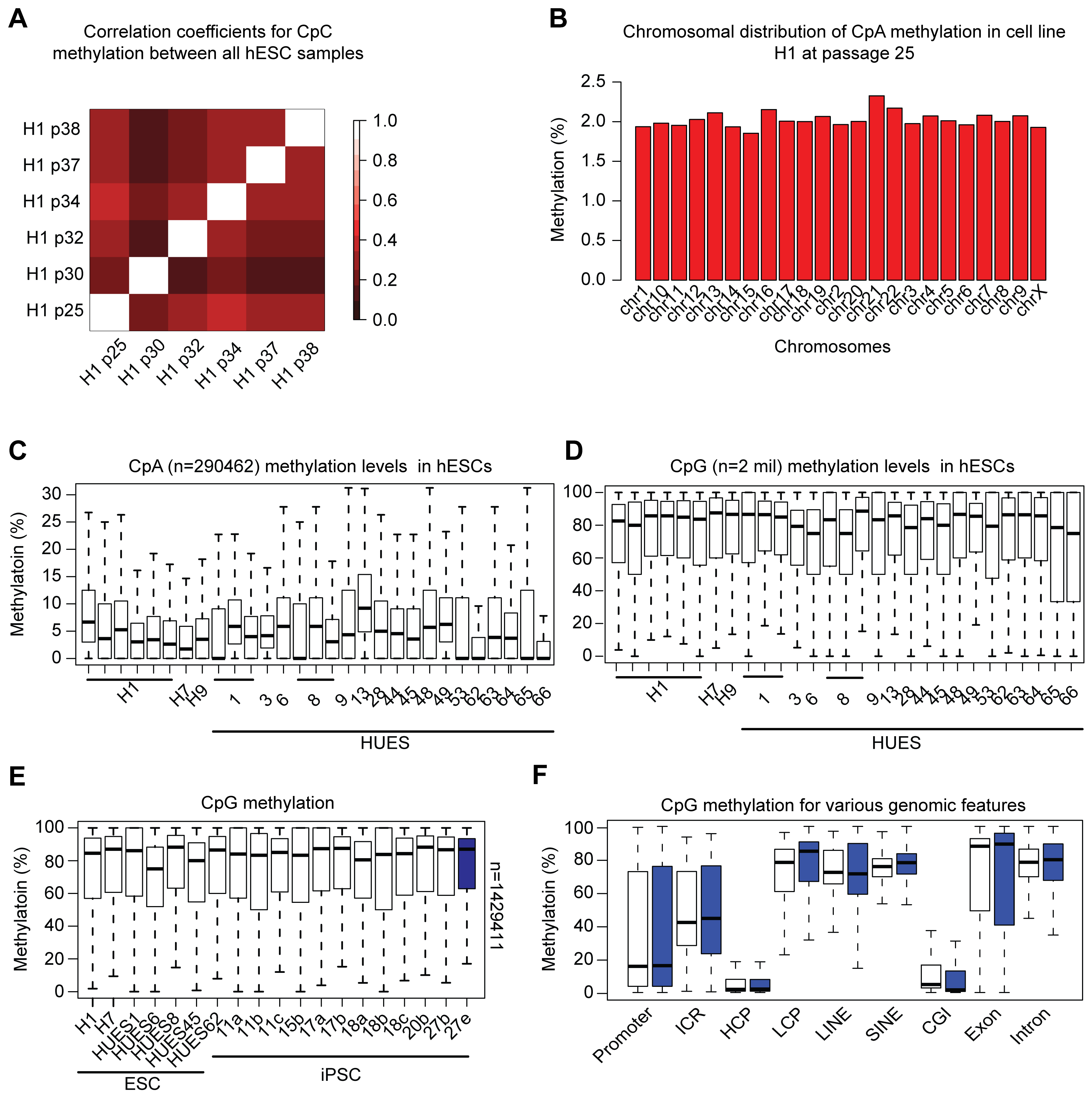

Supplement: Figure S3 — Distribution of non-CpG and CpG methylation in pluripotent cells. (A) Pearson correlation coefficients of individual CpC dinucleotide methylation levels in six replicates of H1. (B) Overall chromosomal distribution of CpA methylation levels in H1p25. (C) Boxplots show CpA methylation levels for all ESC samples on a set of 290,462. CpAs with coverage of at least 5x in more than 80% of all ESC samples and median methylation of ≥0.1%. Boxes are 25th and 75th quartiles, whiskers indicate most extreme data point less than 1.5 interquartile range from box and black bar represents the median. (D) Boxplots show CpG methylation levels for all ESC samples on a set of 2 million CpGs with coverage of at least 5x in more than 80% of all ES cell samples. Boxplots are defined as in C. (E) Distribution of CpG methylation levels in 12 iPSC lines and 7 ESC lines as a reference. Boxplots are based on ∼1.4 million CpGs that show more than 0.1% median methylation levels of 0.1% in the representative ES cell lines (n = 7). Blue boxes indicate samples with the two highest CpA methylation levels relative to the average over all pluripotent cell lines. Boxplots are defined as in C. (F) CpG methylation levels in different genomic region classes in ESC line H1 (p25, p30, p34, p37 and p38; n = 6, white) and iPSC lines 11a, 27e showing high overall CpA methylation levels (n = 2, blue). Genomic features are defined in the Materials and Methods. (TIF) [file pgen.1002389.s003.tif]

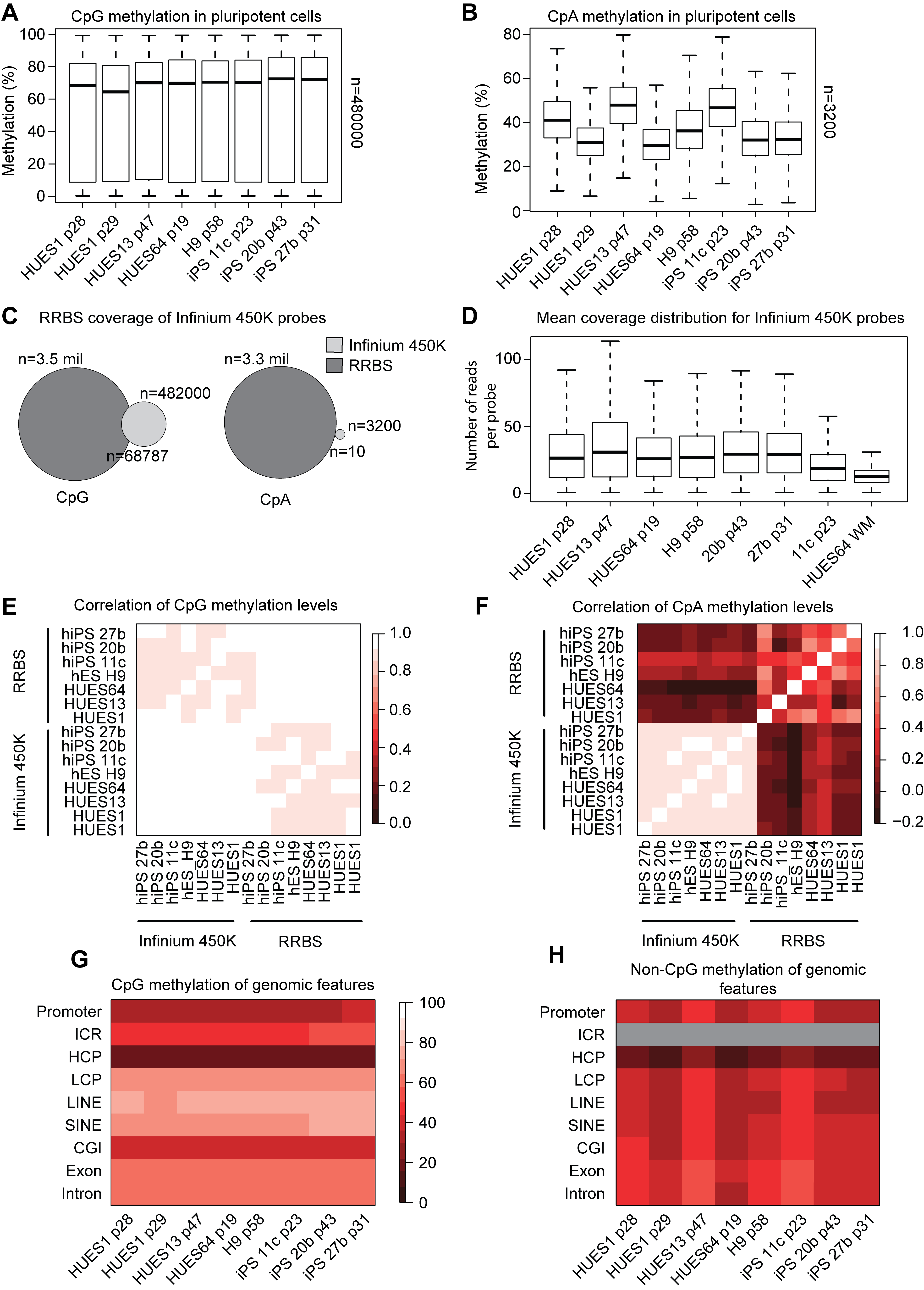

Supplement: Figure S4 — Analysis of methylation in pluripotent cells using the Illumina Infinium 450K array. (A) Distribution of CpG methylation levels in ESCs and iPSCs. Boxes are 25th and 75th quartiles, whiskers indicate most extreme data point less than 1.5 interquartile range from box and black bar represents the median. (B) Distribution of CpA methylation levels in ESCs and iPSCs. Boxes are defined as in A. (C) The venn diagrams show the CpG and CpA dinucleotides covered by RRBS and Infinium 450 K array based on a 40–260 bp size selection. (D) RRBS read coverage distribution for matching samples profiled by Infinium 450 K and RRBS as well as HUES64 WM data. Boxes are defined as in A. (E) Heatmap showing pearson correlation coefficients of CpG methylation levels for matching pluripotent samples based on regions harboring CpGs covered by both RRBS and Infinium 450 K. (F) Heatmap showing pearson correlation coefficients of CpA methylation levels for matching pluripotent samples based on regions harboring CpAs covered by both RRBS and Infinium 450 K. (G) CpG methylation levels of various genomic features according to the Infinium 450 K array. (H) CpA methylation levels of various genomic features according to the Infinium 450 K array. (TIF) [file pgen.1002389.s004.tif]

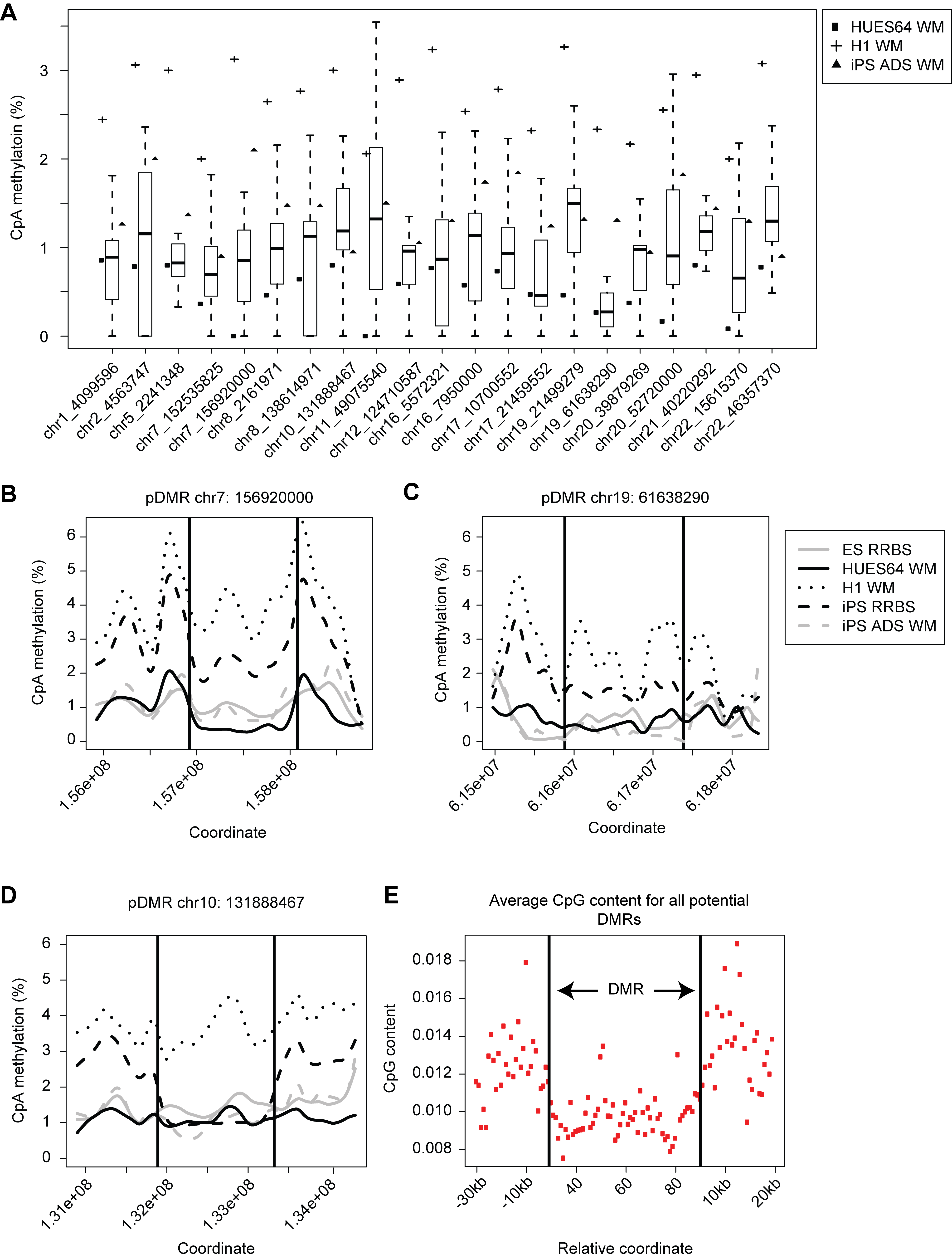

Supplement: Figure S5 — ESCs and iPSCs show no consistent differences in putative DMR regions. (A) CpA methylation levels for 21 putative DMRs reported by Lister et al. 2011 using 20 ESCs (RRBS, boxplots) as a reference, HUES64 WM as well as previously published H1 and iPS ADS WM data [9], [11]. A. (B–D) CpA methylation profile of selected DMRs (framed by black lines) reported by Lister et al. 2011 based on a 1 kb tiling. The CpA methylation levels based on RRBS are shown for the median of all ESCs (n = 20) and all iPSCs (n = 12) as well as for H1p25 WM, iPS ADS WM and HUES64 WM. Regions were selected based on sufficient RRBS coverage (see Materials and Methods). (E) CpG density averaged over all putative DMRs based on a 100 bin tiling for each region. Black bars indicate start and end of putative DMRs. (TIF) [file pgen.1002389.s005.tif]

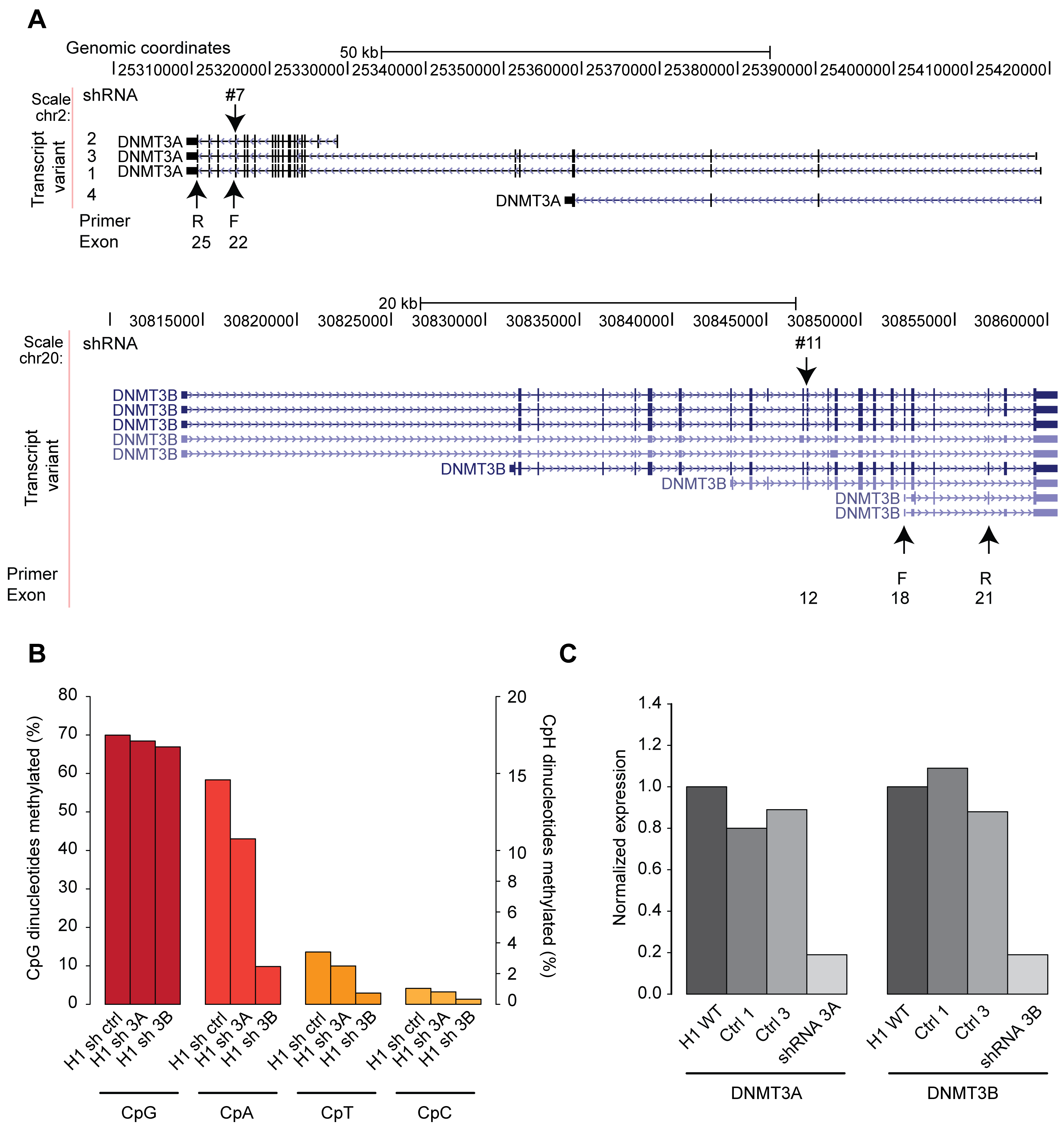

Supplement: Figure S6 — Knockdown of DNMT3A and DNMT3B. (A) Location of PCR primers and shRNA target region in the DNMT3A and DNMT3B gene. (B) Percentage of methylated (≥10%) cytosine dinucleotides in H1 treated with shRNAs against DNMT3A, DNMT3B and control samples. (C) qRT-PCR of DNMT3A in H1 WT, H1 infected with shRNAs against DNMT3A, DNMT3B and control shRNA against GFP. Expression values are normalized to β-Actin levels. (TIF) [file pgen.1002389.s006.tif]

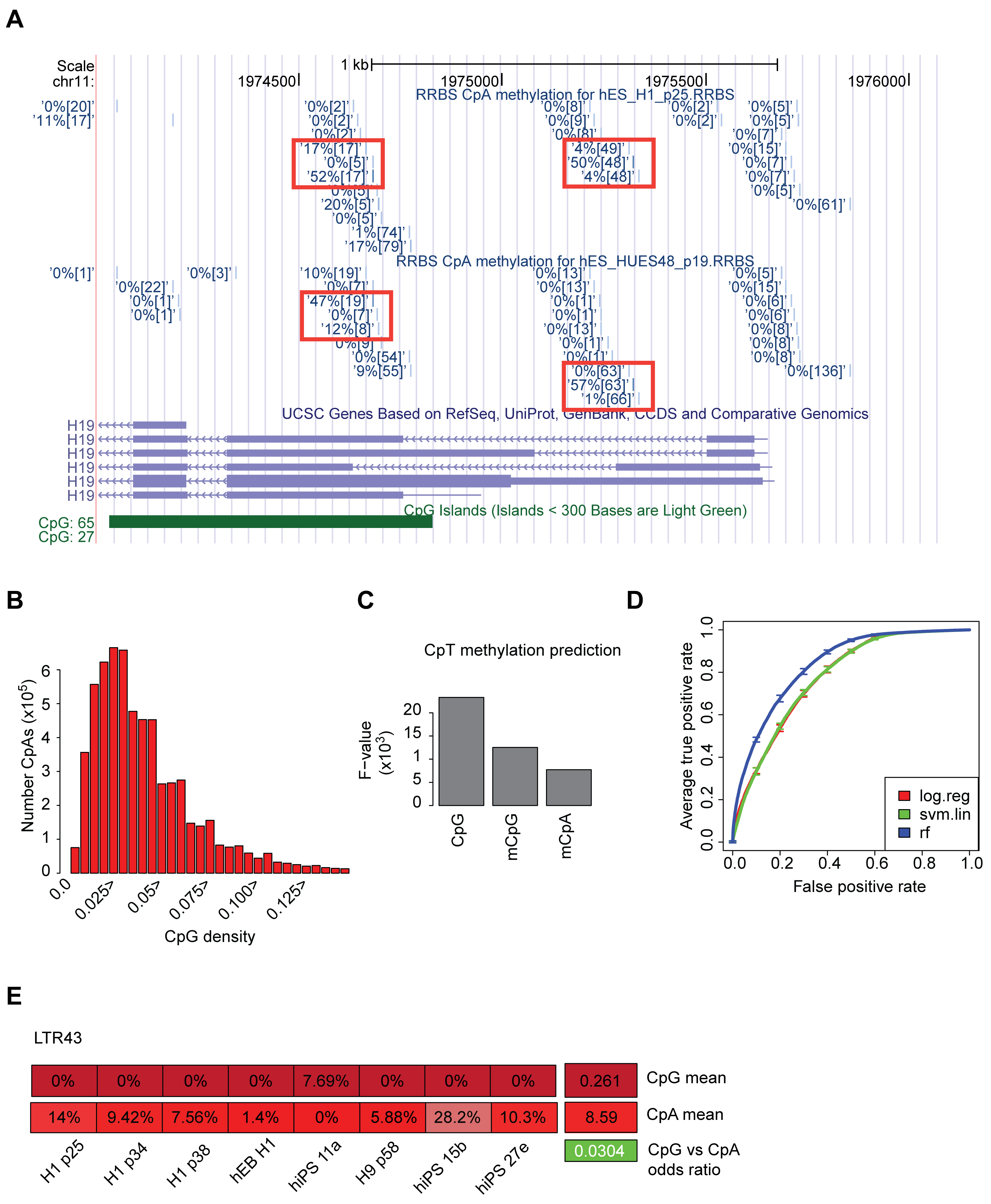

Supplement: Figure S7 — The bona fide DNMT3A target region upstream of H19 shows high CpA methylation levels. (A) Spatial distribution of CpA methylation levels for two ESC lines upstream of the H19 locus. (B) Number of CpAs associated as a function of CpG density based on a genome wide 1 kb tiling. (C) Feature ranking for linear model predicting CpT methylation levels based on ANOVA Only the three most significant features are shown (p-value≤0.000187). Same feature combination as for Figure 5A, 5B was used (Materials and Methods). F-statistics was computed on 9 and 32291 degrees of freedom. (D) Repeat class LTR43 showing the highest CpA methylation levels observed while exhibiting extremely low CpG density. Mean CpG (top) and CpA (bottom) methylation levels obtained from aligning RRBS reads to a pseudogenome consisting prototypic repeat elements (RepBase Update) [19] are shown for 8 representative samples. Coloring corresponds to methylation level (dark red: unmethylated, light red: methylated). Labels in boxes represent percentage of methylation and read covereage. To the right, mean methylation levels across the 8 samples are given along with their odds ratio. (E) ROC curves of three machine learning methods to classify CpA methylation levels. mean AUCs across 10-fold cross-validation was 0.78 for logistic regression and linear support vector machine prediction and 0.83 for random forests. Error bars represent standard deviations. log.reg: logistic regression, smv.lin: linear SVM, rf: random forest. (TIF) [file pgen.1002389.s007.tif]
